# Identifying a Comprehensive ceRNA Network to Reveal Novel Targets for the Pathogenesis of Parkinson's Disease

**DOI:** 10.3389/fneur.2020.00810

**Published:** 2020-08-04

**Authors:** Xi Zhang, Shengyu Feng, Yu Fan, Yuping Luo, Lingjing Jin, Siguang Li

**Affiliations:** ^1^Stem Cell Translational Research Center, Tongji Hospital, Tongji University School of Medicine, Shanghai, China; ^2^Department of Neurology, Tongji Hospital, Tongji University School of Medicine, Shanghai, China; ^3^Key Laboratory of Spine and Spinal Cord Injury Repair and Regeneration of Ministry of Education, Orthopedic Department of Tongji Hospital, Tongji University School of Medicine, Shanghai, China

**Keywords:** Parkinson's disease (PD), competitive endogenous RNAs, ceRNA network, pathogenesis, long non-coding RNAs (lncRNAs)

## Abstract

Parkinson's disease (PD) is the second commonest progressive neurodegenerative disease worldwide. Increasing evidence reveals that non-coding RNAs play roles in the pathophysiological process of PD. The notion called competing endogenous RNAs (ceRNAs) network is used to describe the roles of non-coding RNAs. According to this theory, long non-coding RNAs (lncRNAs) act as microRNAs (miRNAs) sponges by miRNA response elements or miRNA binding sites to control the availability of endogenous miRNA for binding to their target mRNAs. This study aimed to construct a ceRNA network in PD, which might have the potential to clarify the pathogenesis of PD. We investigated differential expression (DE) lncRNAs and mRNAs in substantia nigra array data GSE7621 between PD patients and healthy controls from the Gene Expression Omnibus database. And we used starBase 2.0 and miRWalk 2.0 databases to predict miRNAs that have interactions with DElncRNAs and DEmRNAs. Based on DElncRNAs, DEmRNAs and predicted miRNAs, two ceRNA networks were constructed. The first one was based on lncRNA-miRNA interactions and miRNA-mRNA interactions that shared the same miRNAs that we predicted, on which function annotation and PPI analysis were performed to identify hub genes. Hereby the second ceRNA network was generated to explore the core section in the first ceRNA network and was validated in external datasets. As a result, we identified 31 DE lncRNAs and 1,828 DEmRNAs, and finally constructed the first ceRNA network associated with PD, including 9 lncRNAs, 18 miRNAs, and 185 mRNAs. mRNAs in the first ceRNA network focused on autophagy, DNA repair and vesicle transport, which were critical pathological processes in PD. Nineteen hub genes in the first ceRNA network identified through PPI analysis, the second ceRNA network was constructed to annotate the core part of the first one. Moreover, the core subnetwork was validated in external datasets, of which several nodes including FBXL7, PTBP2, and lncRNA NEAT1 were verified. In conclusion, a ceRNA network was constructed based on the differential expression profiles of whole substantia nigra tissues of normal and PD patients, and the network was subsequently identified which revealed its association with autophagy, DNA repair and vesicle transport. The core subnetwork of the ceRNA network was identified and validated in external data. Our findings offered novel insights into the roles of ceRNAs in the pathogenesis of PD and provided promising diagnostic biomarkers.

## Introduction

Parkinson's disease (PD) is the second commonest progressive neurodegenerative disease worldwide and is characterized by clinically significant non-motor features like constipation, dyssomnia and the classical motor features such as bradykinesia, resting tremor and postural instability (1). The occurrence and development of PD seem to result from a complicated interaction between genetic and environmental factors affecting multiple cellular processes (2). With in-depth research accumulating, numerous PD-susceptibility genes have been identified such as α-synuclein (SNCA), leucine-rich repeat kinase 2 (LRKK2), PTEN induced kinase 1 (PINK1), Parkin and DJ-1, all of which are protein-coding genes (3). However, protein-coding regions make up only 1.5–2% of the human genome, and the remaining are non-coding sequences whose transcripts without protein-coding capacity are named non-coding RNAs (ncRNAs). ncRNAs play important roles in cellular function and disease occurrence which have emerged as a promising field over the years (4). Increasing evidence has emphasized the vital roles of ncRNA in numerous brain biological processes and neurodegenerative disorders (5).

ncRNAs classified by their size including long non-coding RNAs (lncRNAs) and small ncRNA such as miRNAs are now regarded as key regulators involved in gene transcription at the post-transcriptional level which exert their effects on the occurrence, development, and diagnosis of PD (6). For instance, it has been shown that miRNAs (e.g., miR-153 and miR-7) could regulate α-synuclein accumulation that affects the formation of Lewy bodies, the typical characteristic of PD (7); miR-34b and miR-34c could control DJ-1 and Parkin, respectively (8); and lncRNA NaPINK1 can stabilize the expression of PD-susceptibility gene PINK1 in neurons (9).

A hypothesis of how ncRNAs work has been proposed and gradually confirmed. According to this theory called competing endogenous RNAs (ceRNAs) network, lncRNAs act as miRNA sponges by miRNA response elements (MRE) or miRNA binding sites to control the availability of endogenous miRNA for binding to their target mRNAs, which can form a ceRNA network to modulate mRNA expression and regulate protein levels (10). These complex networks may provide multiple clues for unraveling the pathogenesis of PD. The demonstration of lncRNA-associated ceRNA networks in PD might also have the potential as novel therapeutic targets.

**Figure 1:**
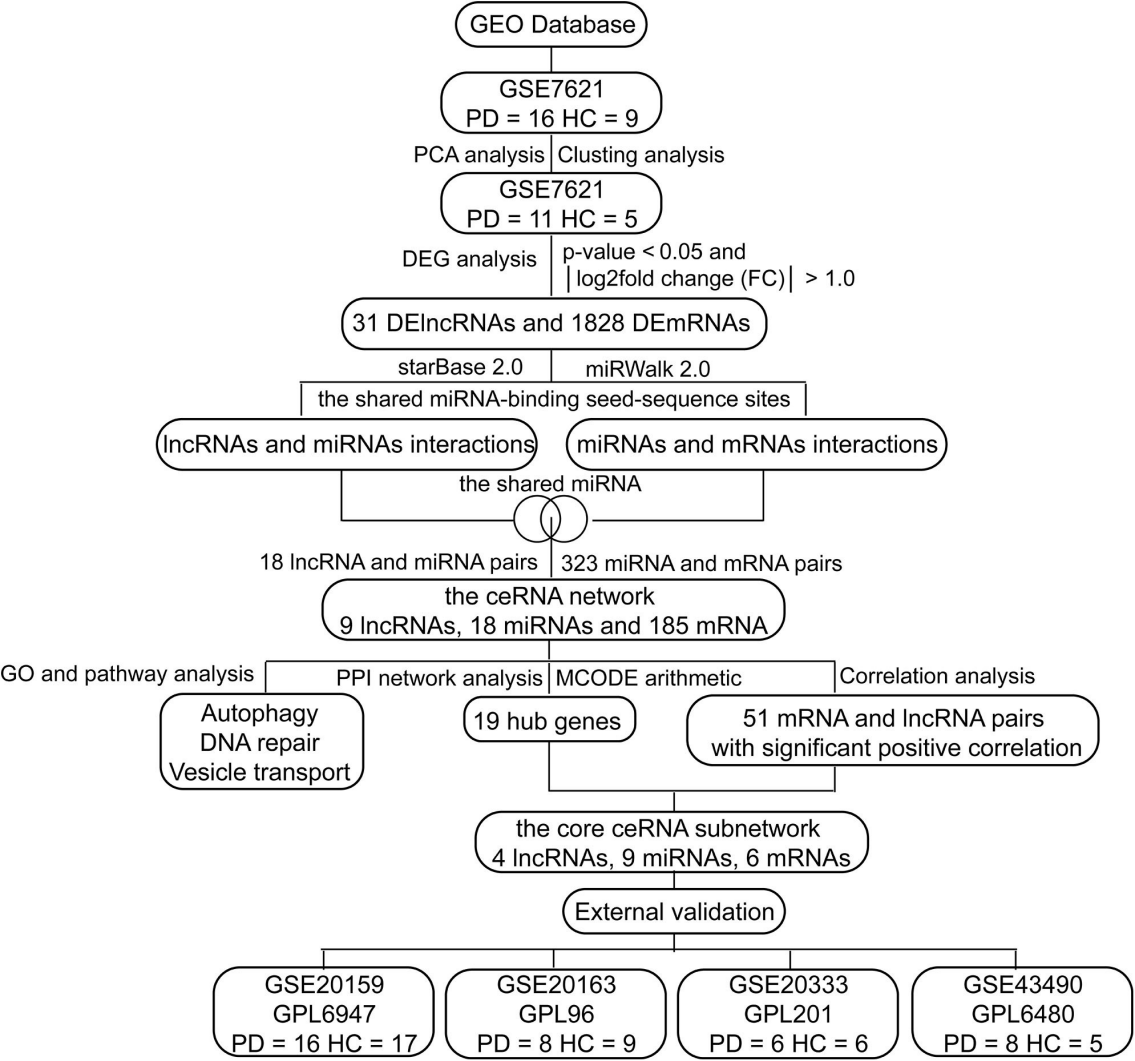
The flow chart of this study.

In this study, we investigated differences in whole substantia nigra RNA expression data between PD patients and healthy controls (HCs) from the National Center of Biotechnology Information (NCBI) Gene Expression Omnibus (GEO) database, and constructed a ceRNA network associated with PD, including 9 lncRNAs, 18 miRNAs and 185 mRNAs (Figure 1). These candidate genes involved in the ceRNA network may serve as promising diagnostic biomarkers for PD.

## Materials and Methods

### Differential Expression Analysis of Gene and lncRNA in PD

**Figure 2:**
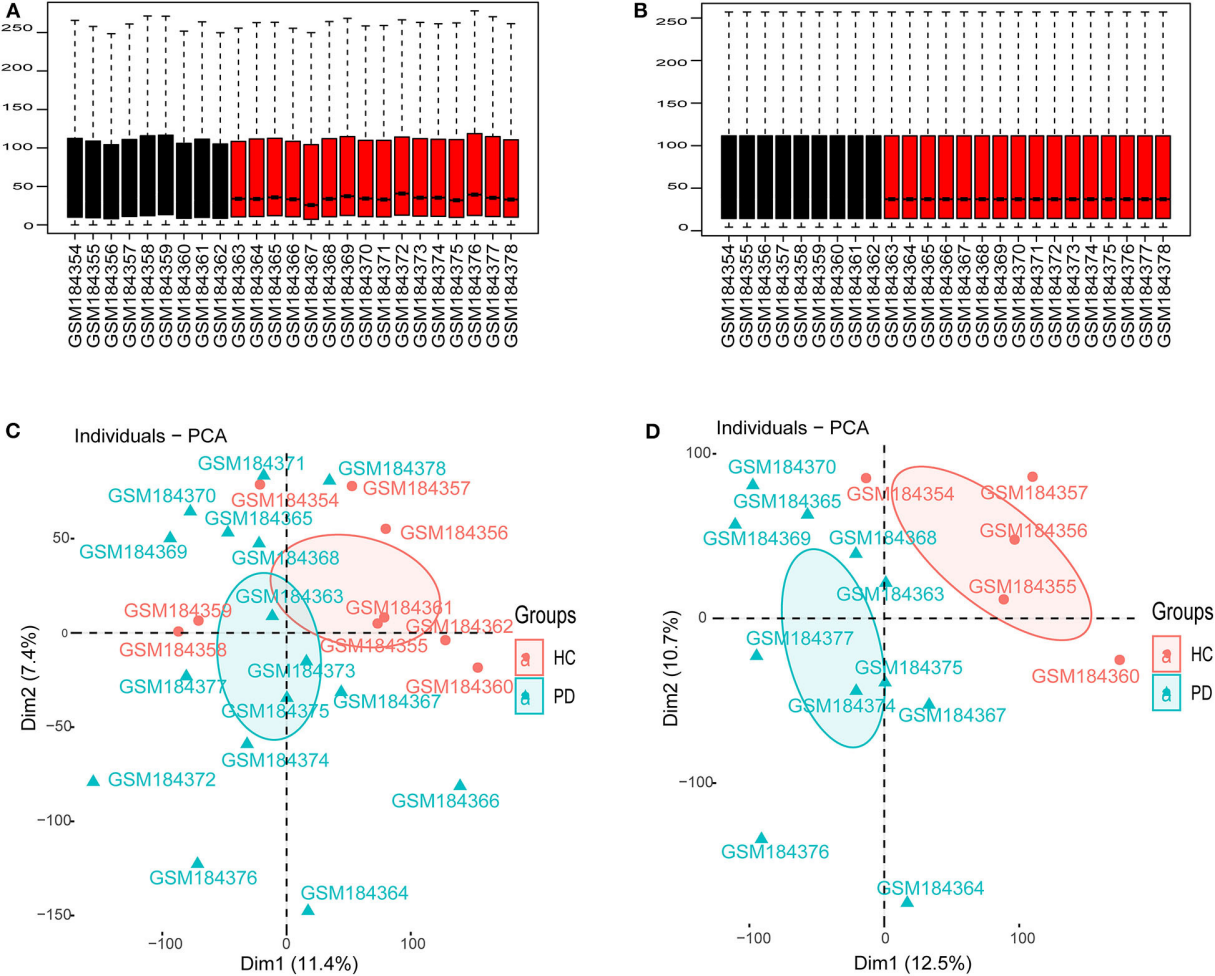
Data preprocessing. **(A)** Boxplot for GSE7621 before quantile normalization. **(B)** Boxplot for GSE7621 after quantile normalization. **(C)** The principal component analysis (PCA) for 16 PD patients and 6 HCs. **(D)** The PCA for selected 11 PD patients and 5 HCs.

As shown in Figure 1, we performed the retrieval in the GEO database with key words “(Parkinson's disease) AND (substantia nigra) AND (human being)” in December 2019, and found that the retrieved datasets were array-based data with different annotation platforms and there were no pure lncRNA datasets. Since the brain-derived lncRNA expression data of PD were unavailable in the GEO database, we compared these platforms and chose GPL570 platform that had the most lncRNA information. Finally, we retrieved the database with the key words “(Parkinson's disease) AND (substantia nigra) AND (human being) AND GPL570.” GSE7621 including mRNAs and numbers of lncRNAs information was obtained and annotated by the GPL570 Platform, Affymetrix Human Genome U133 Plus 2.0 Array. GSE7621 contained whole substantia nigra tissues from postmortem brains of 9 HCs and 16 PD patients. The following parameters were performed under the R environment (version 3.5.3; https://www.r-project.org/), including missing value imputation (If the relative expression levels of certain probe in every samples were zero, this probe was not analyzed), log_2_ transformed [the relative expression level of the probe = log_2_(the value of the probe + 0.001)], background adjustment, quantile normalization (Figures 2A,B), and principal components analysis (PCA) (11). Then we calculated the variation coefficient of all genes, selected the first 5,000 genes with high variation coefficient and performed clustering analysis with these 5,000 genes. According to the cluster analysis and the PCA result (Figures 2C,D, Supplementary Figure 1), we removed nine samples affecting classification and reserved 11 PD patients and 5 HCs. Expression differences were analyzed using the Bayesian analysis provided by the limma package (12) (version 3.42.0, https://bioconductor.org/packages/release/bioc/html/limma.html). Genes with *p* < 0.05 and |Log_2_fold change (FC)| > 1.0 were considered as significantly differential expressed mRNAs (DEmRNAs) and DElncRNAs.

### Construction of lncRNA–miRNA–mRNA Competitive Network

Due to the lack of miRNA information, we predicted miRNAs through online databases. StarBase 2.0 database (13) and miRWalk 2.0 database can provide comprehensive prediction and experimental validation of lncRNA-mRNA interactions and miRNA-mRNA interactions, respectively. We used starBase 2.0 (13) (http://starbase.sysu.edu.cn/starbase2/index.php) and miRWalk 2.0 (14) (http://zmf.umm.uni-heidelberg.de/apps/zmf/mirwalk2/) to predict lncRNA-mRNA interactions and miRNA-mRNA interactions by the shared miRNA-binding seed-sequence sites. The same miRNA-binding sites in both lncRNAs and mRNAs indicated potential lncRNA-miRNA-mRNA interactions. Then we selected lncRNA-mRNA interactions and miRNA-mRNA interactions that shared the same miRNAs to construct the ceRNA network, and visualized it by Cytoscape software (15) (version 3.7.1, http://cytoscape.org).

### Functional Annotation of DEGs in the ceRNA Network

To reveal the function of the ceRNA network, mRNAs in the ceRNA network were subjected to functional enrichment analyses via gene ontology (GO), Kyoto Encyclopedia of Genes and Genomes (KEGG) pathways and Reactome pathways analysis with the R package clusterProfiler (16) (version 3.14.0, https://bioconductor.org/packages/release/bioc/html/clusterProfiler.html) and ReactomePA (17) (version 1.30.0, https://bioconductor.org/packages/release/bioc/html/ReactomePA.html). GO and pathway analyses can be the translation of the list of differentially expressed genes into a functional list, and statistically highlight the most over-represented (enriched) functional terms out of a list of hundreds or thousands of terms. These can offer insight into the cellular mechanisms relevant in the given condition (18). The significant selection criteria were *p* < 0.01 and gene count ≥ 6.0. The enrichment analyses were visualized by the R package GOplot (19) (version 1.0.2, https://cran.r-project.org/web/packages/GOplot/).

### Integration of Protein-Protein Interaction (PPI) Network and Construction of the Core ceRNA Subnetwork

To explore the core genes, we performed PPI network analysis and clustering analysis called MCODE based on all mRNAs in the ceRNA network. The Search Tool for the Retrieval of Interacting Genes (STRING) online tool (20) (http://string-db.org/) was applied to analyze the PPI of DEmRNAs in the ceRNA network with the threshold of combined score >0.40. The PPI network was constructed using the Cytoscape based on the obtained PPI relationships. The highly interconnected modules in the PPI network were analyzed using the plug-in MCODE (21) with the cut-off score >5.0. To estimate the correlations of mRNAs and lncRNAs in the ceRNA network, the correlation analysis was performed with the following significant selection criteria, including Pearson correlation coefficient *r* > 0.04 and *p* < 0.05. The core ceRNA subnetwork was constructed according to the hub genes and their associated lncRNAs.

### Data Verification in Other Independent Datasets

To verify the ceRNA subnetwork and our results, we identified DEGs of GSE20159, GSE20163, GSE20333, and GSE43490, respectively, which included lncRNAs and mRNAs information of PD patients and HCs. We analyzed these datasets with the same processes and selection criteria as those of GSE7621. If the relative expression levels of genes in the ceRNA network were significantly differential in two or more datasets simultaneously, we considered that these genes are validated.

## Results

### 31 DElncRNAs and 1,828 DEmRNAs Were Identified Between PD Patients and HCs

**Figure 3:**
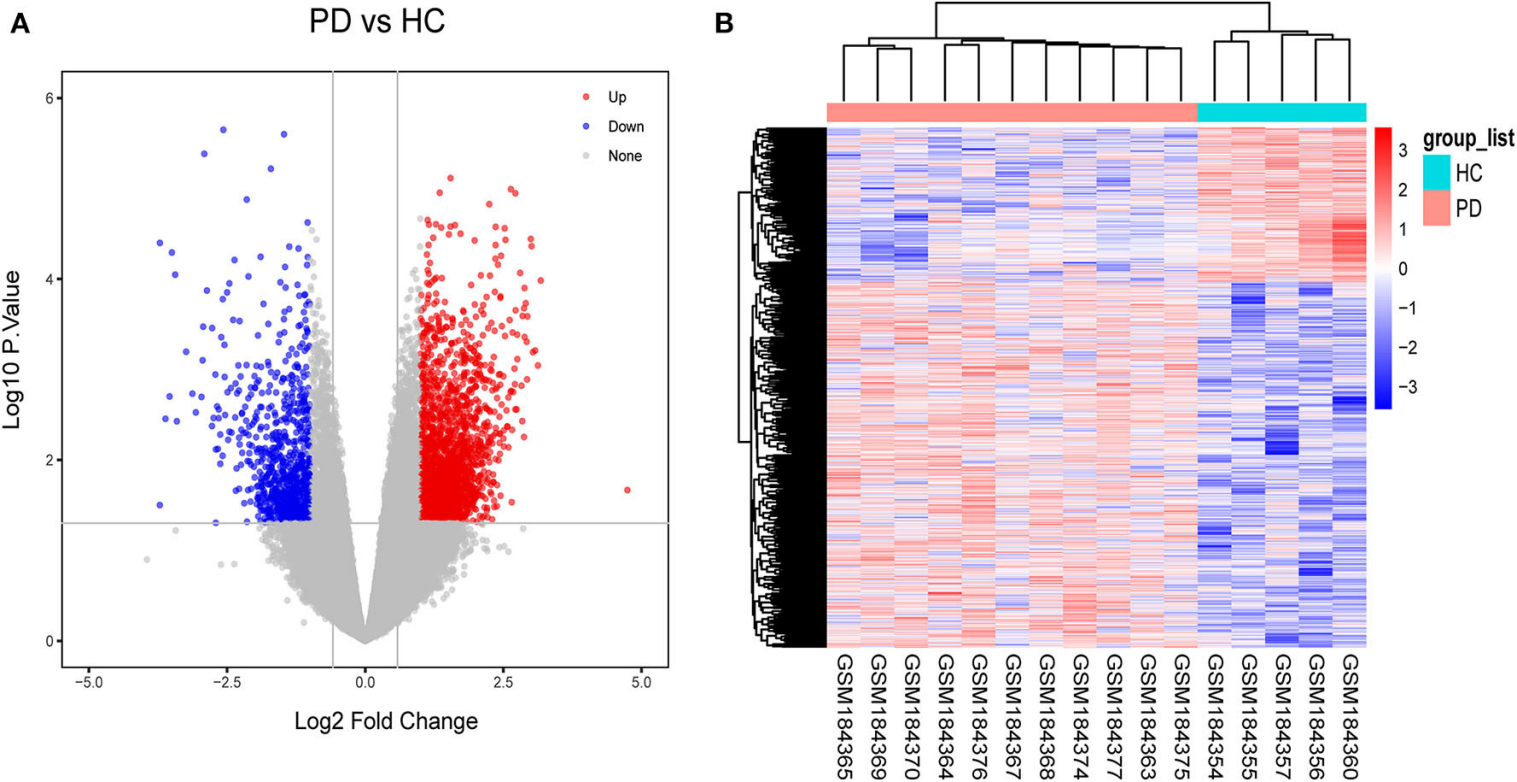
Identification of gene expression profile about 11 PD patients and 5HCs. **(A)** Volcano plot of microarray data. **(B)** The cluster heat map of differential expression genes (DEGs).

As a result, 31 DElncRNAs and 1,828 DEmRNAs were identified except miRNA due to the limitation of the array (Figure 3A). The heatmap of the DElncRNAs and DEmRNAs showed PD patients could be distinguished from HCs (Figure 3B). In general, when healthy samples were used as controls, we identified 31 DElncRNAs (11 downregulated and 20 upregulated) and 1,828 DEmRNAs (741 downregulated and 1,084 upregulated) in PD patients, which provide the possibility for constructing ceRNA networks about PD.

### Construction of lncRNA-Associated ceRNA Networks About PD

**Figure 4:**
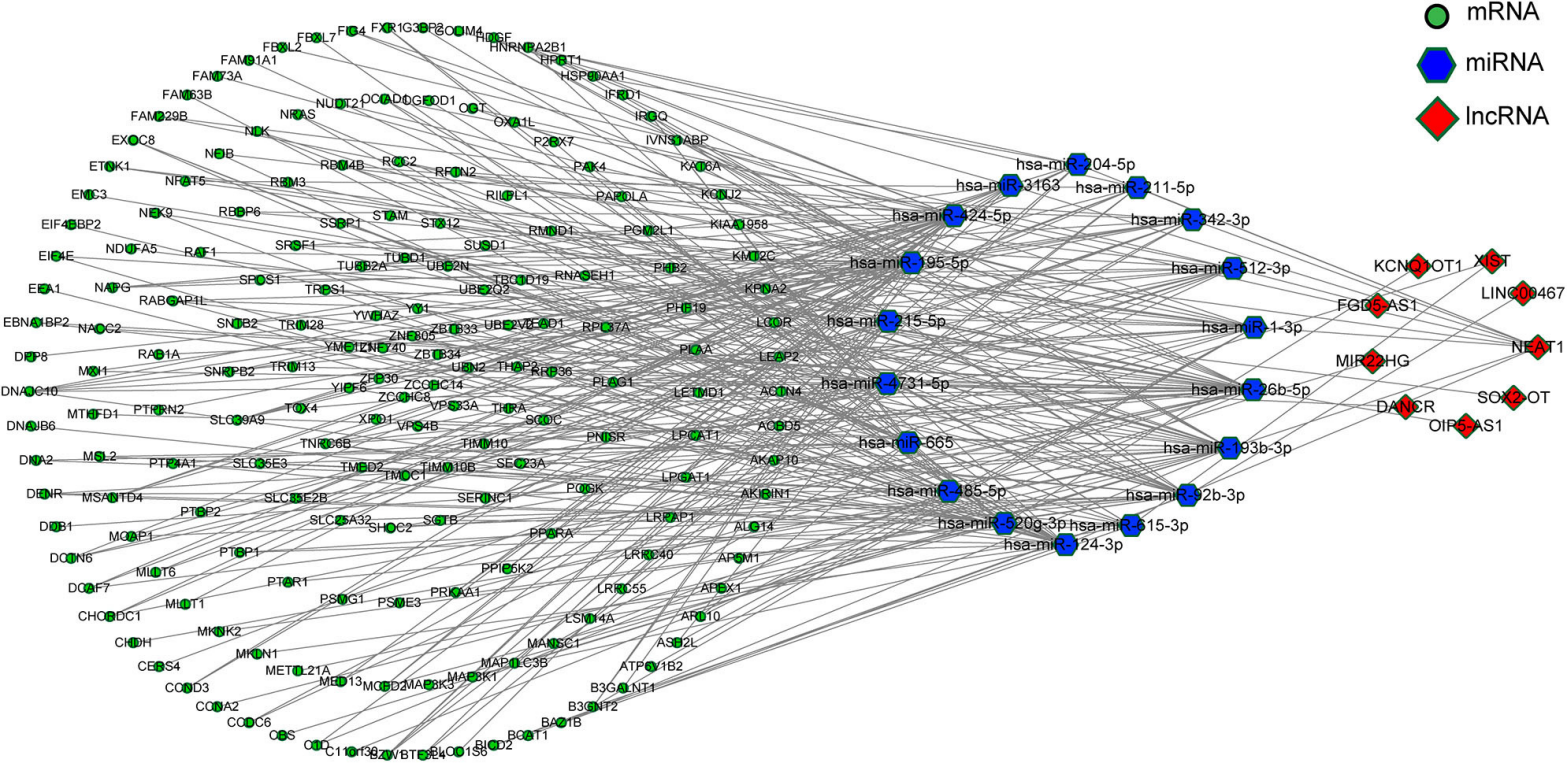
The ceRNA network.

According to the ceRNA hypothesis, the ceRNA crosstalk was identified by the shared miRNAs between lncRNAs and mRNAs. 18 miRNA-lncRNA and 323 miRNA-mRNA interactions were obtained and a ceRNA network was constructed including 341 edges and 212 nodes (including 9 lncRNAs, 18 miRNAs, and 185 mRNA) (Figure 4). These RNA interactions might be pivotal for the pathogenesis of PD.

### mRNAs in the ceRNA Network Were Enriched in Autophagy, DNA Repair and Vesicle Transport

**Figure 5:**
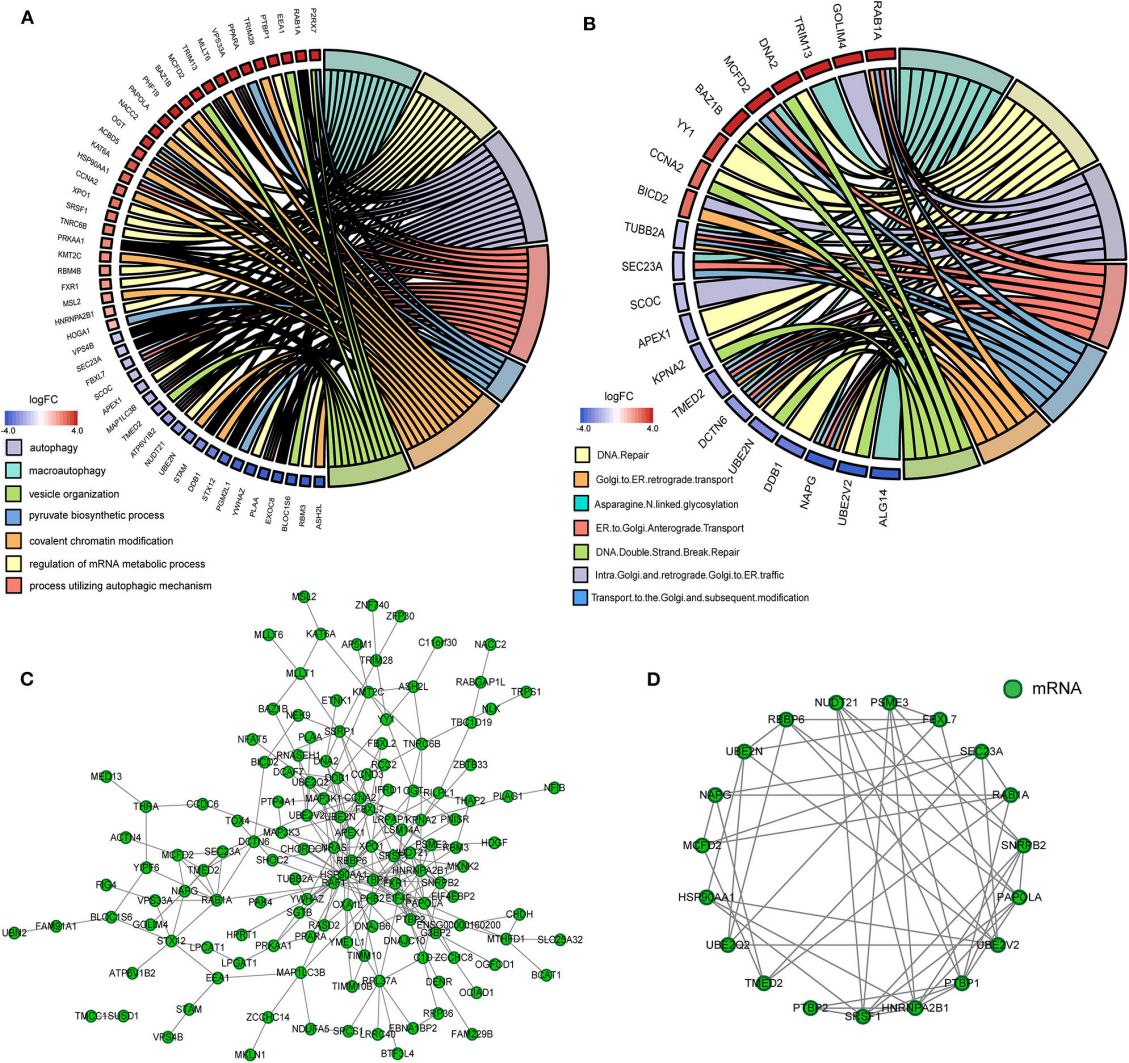
The functional annotation and protein-protein interaction (PPI) analysis of all mRNAs in the ceRNA network. **(A)** The circos plot of significant gene ontology analyses enriched by mRNAs in the ceRNA network. **(B)** The circos plot of significant Reactome pathways enriched by mRNAs in the ceRNA network. **(C)** The PPI network of mRNAs in the ceRNA network. **(D)** Cluster 1 analyzed by the plug-in MCODE in the whole PPI network.

As the result of GO based on mRNAs in the ceRNA network (Figure 5A), 7 GO terms were significantly enriched (Table 1). According to the pathway analyses (Figure 5B), there are 7 significant Reactome pathways (Table 2). In general, these functional annotations show the key biological processes and the critical signaling cascades including autophagy (macroautophagy, autophagy, a process utilizing autophagic mechanism), DNA repair (DNA repair, DNA double-strand break repair) and vesicle transport (vesicle organization, intra-Golgi and retrograde Golgi-to-ER traffic, ER to Golgi anterograde transport, transport to the Golgi and subsequent modification, Golgi-to-ER retrograde transport), all of which participate in the pathogenesis of PD.

**Table 1:** The significant gene ontology terms.

**ID**	**Description**	***p*.adjust**	**Count**	**geneID**
GO:0016236	Macroautophagy	2.18E-06	13	ATP6V1B2/EXOC8/MAP1LC3B/PLAA/PRKAA1/RAB1A/SCOC/SEC23A/STAM/STX12/TRIM13/VPS33A/VPS4B
GO:1903311	Regulation of mRNA metabolic process	6.99E-06	12	APEX1/FXR1/HNRNPA2B1/NUDT21/PAPOLA/PTBP1/RBM3/RBM4B/SRSF1/TNRC6B/XPO1/YWHAZ
GO:0006914	Autophagy	7.61E-06	16	ACBD5/ATP6V1B2/EXOC8/FBXL2/HSP90AA1/MAP1LC3B/PLAA/PRKAA1/RAB1A/SCOC/SEC23A/STAM/STX12/TRIM13/VPS33A/VPS4B
GO:0061919	Process utilizing autophagic mechanism	7.61E-06	16	ACBD5/ATP6V1B2/EXOC8/FBXL2/HSP90AA1/MAP1LC3B/PLAA/PRKAA1/RAB1A/SCOC/SEC23A/STAM/STX12/TRIM13/VPS33A/VPS4B
GO:0042866	Pyruvate biosynthetic process	8.76E-05	6	HOGA1/OGT/P2RX7/PGM2L1/PPARA/PRKAA1
GO:0016569	Covalent chromatin modification	0.000132	14	ASH2L/BAZ1B/CCNA2/DDB1/KAT6A/KMT2C/MLLT6/MSL2/NACC2/OGT/PHF19/PRKAA1/TRIM28/UBE2N
GO:0016050	Vesicle organization	0.000177	11	BLOC1S6/EEA1/EXOC8/MCFD2/P2RX7/RAB1A/SEC23A/STAM/STX12/TMED2/VPS4B

**Table 2:** The significant reactome pathways.

**ID**	**Description**	***p*-value**	**Count**	**geneID**
R-HSA446203	Asparagine N-linked glycosylation	0.005	9	ALG14/DCTN6/MCFD2/NAPG/RAB1A/SEC23A/TMED2/TRIM13/TUBB2A
R-HSA-73894	DNA Repair	0.007	9	APEX1/BAZ1B/CCNA2/DDB1/DNA2/KPNA2/UBE2N/UBE2V2/YY1
R-HSA-6811442	Intra-Golgi and retrograde Golgi-to-ER traffic	0.001	8	BICD2/DCTN6/GOLIM4/NAPG/RAB1A/SCOC/TMED2/TUBB2A
R-HSA-199977	ER to Golgi Anterograde Transport	0.001	7	DCTN6/MCFD2/NAPG/RAB1A/SEC23A/TMED2/TUBB2A
R-HSA-948021	Transport to the Golgi and subsequent modification	0.003	7	DCTN6/MCFD2/NAPG/RAB1A/SEC23A/TMED2/TUBB2A
R-HSA-8856688	Golgi-to-ER retrograde transport	0.002	6	BICD2/DCTN6/NAPG/RAB1A/TMED2/TUBB2A
R-HSA-5693532	DNA Double-Strand Break Repair	0.009	6	BAZ1B/CCNA2/DNA2/KPNA2/UBE2N/UBE2V2

### Identification of Core Genes and Construction of the Core ceRNA Subnetwork

**Figure 6:**
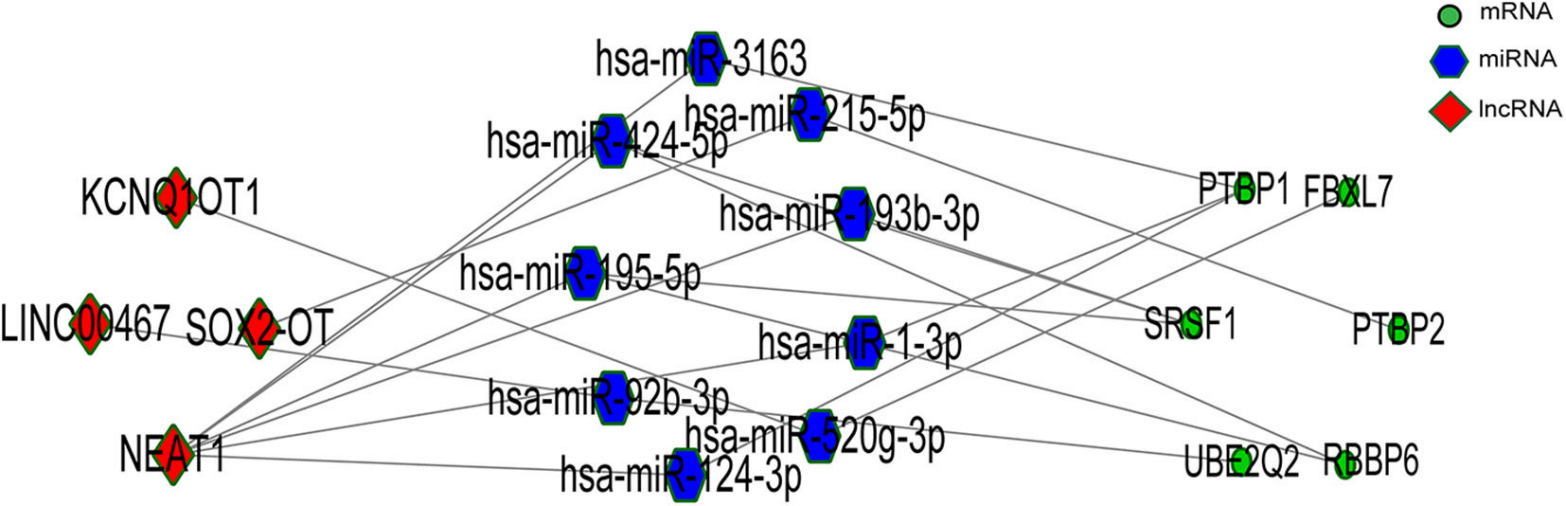
The lncRNA-associated ceRNA network based significant correlations.

We gained 19 hub genes based on the PPI network analysis (Figure 5C) and clustering analysis called MCODE (Figure 5D). According to the ceRNA hypothesis, the expression of lncRNAs might be positively correlated with their regulated mRNAs. Combining the 19 hub mRNAs and 51 lncRNA-mRNA pairs with their corresponding correlations, we constructed the core subnetwork of the ceRNA network, which contained 9 miRNA-lncRNA and 11 miRNA-mRNA interactions, and a total of 19 nodes (including 4 lncRNAs, 9 miRNAs, 6 hub mRNAs) (Figure 6, Table 3). The subnetwork may play key roles in the ceRNA network, providing more core information to clarify the role of the ceRNA network in the pathogenesis of PD.

**Table 3:** The lncRNA-mRNA pairs with significant correlations.

**lncRNA**	**mRNA**	r^*^	***p-value***
KCNQ1OT1	FBXL7	0.613	0.012
NEAT1	PTBP1	0.703	0.002
SOX2-OT	PTBP2	0.634	0.008
NEAT1	RBBP6	0.703	0.002
NEAT1	SRSF1	0.551	0.027
LINC00467	UBE2Q2	0.565	0.023

* *Pearson correlation coefficient*.

### Verification of the ceRNA Subnetwork by GEO Datasets

**Figure 7:**
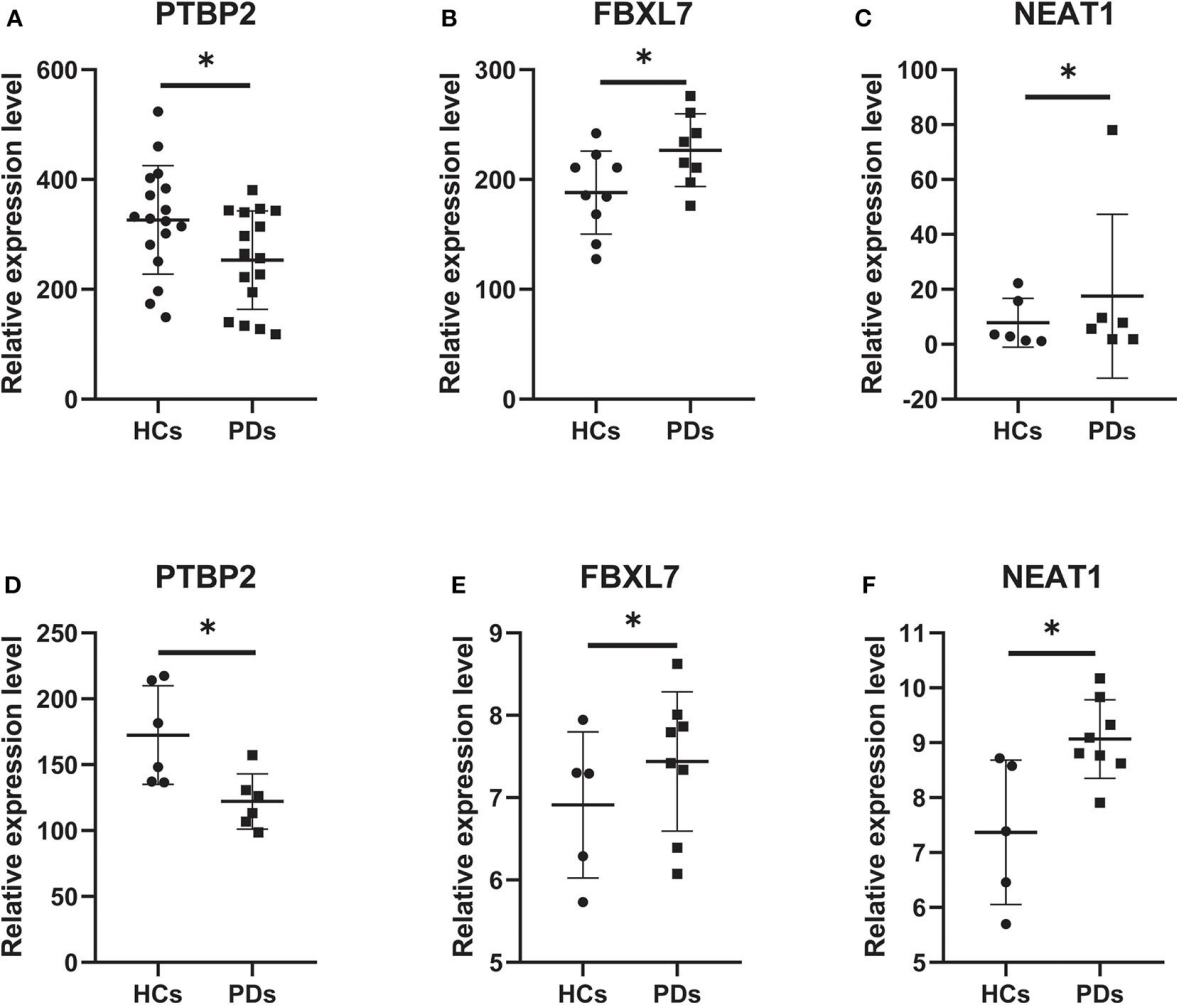
Core genes verified in other GEO datasets. **(A)** The expression level of PTBP2 in GSE20159. **(B)** The expression level of FBXL7 in GSE20163. **(C)** The expression level of NEAT1 in GSE20333. **(D)** The expression level of PTBP2 in GSE20333. **(E)** The expression level of FBXL7 in GSE43490. **(F)** The expression level of NEAT1 in GSE43490. Data are presented as means ± SD (*p < 0.05).

According to the external validation based on other datasets, we obtained several genes that were significantly differentially expressed in two or more datasets simultaneously (Figure 7). For example, nuclear paraspeckle assembly transcript 1 (NEAT1) has been already identified as the paraspeckle assembly scaffold. The expression of NEAT1 in the substantia nigra and peripheral blood cells of PD patients was higher than that in HCs (22, 23). NEAT1 was identified as the ceRNA to regulate serine/arginine-rich splicing factor 1 (SRSF1), and this relationship had been verified *in vitro* experiments (24). Combined with datasets and published literatures, the idea that the constructed ceRNA network may play roles in PD has been partially verified. Therefore, we identified the key subnetwork in the constructed ceRNA network which may affect the pathogenesis of PD.

## Discussion

PD is the second commonest neurodegenerative disease with unclear pathogenesis. Although plenty of protein-coding genes have been identified as PD-related genes, these genes couldn't perfectly explain how PD occurs and develops. Recently, a growing body of research has focused on the epigenetic regulation and the roles of ncRNAs in PD pathogenesis (6). In this study, we constructed the ceRNA network based on the expression profiles of whole substantia nigra tissues of healthy controls and PD patients.

Then we performed the functional analyses of GO enrichment and Reactome pathways based on the genes in the ceRNA network and identified not only numerous enriched terms related to PD, including autophagy and vesicles organization, but also plenty of pathways, including DNA Repair and vesicles transport between Golgi and ER. In short, the functional analyses of the ceRNA network focused on three aspects including autophagy, DNA repair and vesicle transport, all of which take part in the pathogenesis of PD (25–27).

To identify the core of ceRNA networks, we gained 19 hub genes by PPI and MCODE analyses. Based on the correlations between hub genes and their paired lncRNAs, we selectively analyzed these pairs with significant positive correlation and constructed a ceRNA subnetwork. For external validation, we investigated the relative expression levels of these nodes in other datasets and verified several genes in the ceRNA subnetwork which are polypyrimidine tract binding protein 2 (PTBP2), F-Box and Leucine-Rich Repeat Protein 7 (FBXL7) and lncRNA NEAT1. PTBP2 is an abundant multifunctional RNA-binding protein implicated in various aspects of cellular mRNA metabolism in the brain and can interact with mitochondrial tRNA to affect mitochondria function (28). FBXL7 overexpression induces mitochondrial injury through affecting the fission of mitochondria and mitochondrial deficiency which is the key organelle in PD pathogenesis (29). NEAT1 affects not only autophagy in MPTP models (30), but also mitochondria. NEAT1 depletion has profound effects on mitochondrial dynamics and functions by altering the sequestration of *mito*-mRNAs (31). LncRNA NEAT1 could be identified as the ceRNA of numerous miRNAs and thus influences plenty of mRNAs participating in the occurrence and development of PD.

Because the existing microarray data cannot completely detect mRNA, miRNA, and lncRNA in samples, the results cannot be completely verified. Nevertheless, other unverified data may also have important roles in PD. For example, there are reports that PTBP1 involved in the stabilization and mRNA translation of insulin can be a longitudinally dynamic biomarker for PD (32), and can be regulated by NEAT1 according to POSTAR2 database (24). Besides, our results also revealed that NEAT1 could be the ceRNA of three miRNAs targeting SRSF1 which has an important role in DNA damages (33), and this relationship had been verified *in vitro* experiments (24).

Previous literature constructed ceRNA networks based on RNA expression data of PD patients' blood samples to explore the role of the ceRNA network in PD, which pointed that XIST and PART1 could be the sponge of hsa-miR-133b and regulate the IGF1R expression (34). Although samples come from blood and brain tissue, it is the same conclusion that lncRNA XIST could be involved in PD through ceRNA networks. Besides, we identified other lncRNAs, especially lncRNA NEAT1 and its related ceRNA networks, which may be more significant.

The biggest limitation of this study is that the GPL570-based microarray data do not contain total lncRNAs and miRNAs. Despite this, the compositions of networks are slightly different. Besides, the sample size of the data was too small even though the external validation made our results more convincing, there was lack of physiological and pathological information to analyze the associations of clinicopathological characteristics and these ceRNA networks, and the biological mechanisms and functions mediated by the ceRNA are still needed to verify *in vivo* in the future.

In conclusion, the substantia nigra lncRNA-associated ceRNA network of PD was constructed. Our findings improve the current understanding of ceRNA biological behaviors and the regulatory roles in the pathogenesis of PD. The novel candidate genes in the networks may serve as promising diagnostic biomarkers and therapeutic targets for PD treatment.

## Data Availability Statement

All datasets presented in this study are included in the article/Supplementary Material.

## Author Contributions

XZ and SF designed the research topic, drafted the manuscript, and analyzed the data. XZ and YF participated in the revision of the manuscript and figures. YL, SL, and LJ were involved in the work instruction and financial support. All authors contributed to the article and approved the submitted version.

## Conflict of Interest

The authors declare that the research was conducted in the absence of any commercial or financial relationships that could be construed as a potential conflict of interest.

## Supplementary Material

Supplementary Figure 1 The clustering result of GSE7621. The samples with red font were the samples we removed, and the samples with black font were the samples we analyzed.

Supplementary Table 1 The gender information of samples in this study.
