# Supplementary material for: Identifying a Comprehensive ceRNA Network to Reveal Novel Targets for the Pathogenesis of Parkinson's Disease
Source: Front Neurol. 2020 Aug 4;11:810. doi: 10.3389/fneur.2020.00810 (PMC7417679; doi:10.3389/fneur.2020.00810)
Supplement: Supplementary Table 1 — The gender information of samples in this study. [file Table_1.docx]

Supplementary Table 1. The gender information of samples in this study

| **Group** | **Sample size before selection** | **Sample size after selection** |
| --- | --- | --- |
| Parkinson’s disease group | 19 | 11 |
| Males | 13 | 9 |
| Females | 3 | 2 |
| Healthy controls group | 9 | 5 |
| Males | 4 | 2 |
| Females | 5 | 3 |
